# probeBase—an online resource for rRNA-targeted oligonucleotide probes and primers: new features 2016

**DOI:** 10.1093/nar/gkv1232

**Published:** 2015-11-19

**Authors:** Daniel Greuter, Alexander Loy, Matthias Horn, Thomas Rattei

**Affiliations:** 1 Division of Computational Systems Biology, Department of Microbiology and Ecosystem Science, Research Network Chemistry meets Microbiology, University of Vienna, A-1090 Wien, Austria; 2 Division of Microbial Ecology, Department of Microbiology and Ecosystem Science, Research Network Chemistry meets Microbiology, University of Vienna, A-1090 Wien, Austria

## Abstract

probeBase http://www.probebase.net is a manually maintained and curated database of rRNA-targeted oligonucleotide probes and primers. Contextual information and multiple options for evaluating *in silico* hybridization performance against the most recent rRNA sequence databases are provided for each oligonucleotide entry, which makes probeBase an important and frequently used resource for microbiology research and diagnostics. Here we present a major update of probeBase, which was last featured in the NAR Database Issue 2007. This update describes a complete remodeling of the database architecture and environment to accommodate computationally efficient access. Improved search functions, sequence match tools and data output now extend the opportunities for finding suitable hierarchical probe sets that target an organism or taxon at different taxonomic levels. To facilitate the identification of complementary probe sets for organisms represented by short rRNA sequence reads generated by amplicon sequencing or metagenomic analysis with next generation sequencing technologies such as Illumina and IonTorrent, we introduce a novel tool that recovers surrogate near full-length rRNA sequences for short query sequences and finds matching oligonucleotides in probeBase.

## INTRODUCTION

Our understanding of the diversity and role of microorganisms on our planet is to a great extent based on exploiting the ribosomal RNA as phylogenetic marker molecule in diagnostic molecular biology and microscopy assays. While rRNA-targeted oligonucleotides have been applied in different sorts of diagnostic formats such as DNA microarrays (PhyloChips) (1,2) and denaturing gradient gel electrophoresis (3), they are now most widely used for amplicon sequencing and fluorescence *in situ* hybridization (FISH) (4). Today, highly multiplexed amplicon sequencing with rRNA-targeted primers enables surveying microbial diversity across numerous samples (5,6), which provides unprecedented insights into the spatial distribution and temporal dynamics of the diverse microbial communities that thrive in the environment (7) or are associated with eukaryotic hosts (8,9). Furthermore, FISH with rRNA-targeted probes and quantitative microscopy is a standard tool for revealing the identity, abundance and spatial localization of microbial cells in complex samples. More than two decades of development of FISH probes and techniques for microbial diagnostics have established a variety of methods, such as DOPE-FISH (10), CARD-FISH (11), CLASI-FISH (12) and HCR-FISH (13), and a wealth of tested probes that target diverse phylogenetic and/or taxonomic groups of microorganisms. probeBase was originally established in 2002 (14) to provide a common, freely accessible repository for rRNA-targeted oligonucleotide sequences, including contextual information and multiple options for testing *in silico* specificity and coverage (15) against up-to-date rRNA sequence databases such as RDP-II (16) and SILVA (17). To date (September 2015), probeBase contains 2788 probes, 175 domain-specific PCR primers (18) and 16 microarrays from 499 publications and is an online resource that is frequently used by the scientific community (180 000 average page views per year).

Finding appropriate oligonucleotides with a suite of ‘Search’ and sequence ‘Match’ tools provides convenient access to the information in the database. Probes, primers, microarray layouts or references can further be retrieved through the ‘Lists’ service, including dynamic lists of all probes, all primers, all references or oligonucleotides that target microorganisms from specific environments (e.g. intestinal microbiota) or with specific functions (e.g. sulfate-reducing microorganisms).

This update describes recent improvements and new features added since the last update in 2007 (19), including (i) extended ‘Search’ and ‘Match’ options, (ii) a new ‘Proxy’ tool that finds probe sets for short query sequences based on corresponding near full-length rRNA sequences and (iii) suggestion of taxonomically informed hierarchical probe sets for applications using multiple probes such as multi-color FISH and DNA microarrays.

## NEW DATABASE BACKEND, SEARCH ENVIRONMENT AND WEBSITE FOR probeBase

The probeBase database has been moved to a new, more scalable database backend. This dramatically reduces the retrieval times when the database is queried and it can also handle a much larger number of requests simultaneously. A new database scheme was developed, which links probeBase entries with the NCBI taxonomy database (20). Database procedures were implemented to retrieve the taxonomic lineages (up and down) of each specificity term instantly from the NCBI taxonomy database. Thereby all changes in the reference taxonomy will be automatically adopted by probeBase.

The continuously growing number of sequences in probeBase and in rRNA sequence databases (16,17) made it necessary to refine the search environment of probeBase. The core of the new search and match tools are sequence indexes based on enhanced suffix array data structures. These suffix arrays allow very short retrieval times by rapid exact string matching. They are used by VMATCH (21) for probe/primer search (‘Search’) and sequence match (‘Match’), and by LAST (22) for the proxy sequence match (‘Proxy’) functionality (see below for a description of the ‘Search’, ‘Match’ and ‘Proxy’ tools). The exact string matching in VMATCH is not aware of DNA ambiguity characters, such as R, Y, W, etc. probeBase therefore refines the alignments calculated by VMATCH for the sequence match (‘Match’) function and considers such ambiguity positions to determine the correct number of mismatches also for these positions.

The probeBase web page has been moved to a new content management system to facilitate maintenance and more rapid adaptations of the web page. Another advantage of the content management system is the responsible layout, which considers the size of the browsing device. Hence, the page will be optimized for smaller displays if users access probeBase via their mobile phone or tablet computer.

Result lists are now fully sortable by just clicking on the header of the respective column. In addition, longer tables are being split into multi-page tables to give the user a convenient overview even if a certain database query returns many results. This feature is particularly important due to the increased number of supported sequences per user query. The multi-page table views are accompanied by an export function. Users are able to export results in .xls and .tsv format, which allows performing further analysis in any other suitable software, such as Excel or OpenOffice.

## A TARGET TAXONOMY FOR EACH OLIGONUCLEOTIDE

Detailed information is provided for each oligonucleotide (14,19), including its specificity, which indicates the intended target organism(s) of the respective probe/primer as described in the original publication or during user submissions to probeBase. Based on the information in the specificity field, we have automatically mapped each oligonucleotide to the NCBI taxonomy (20). Where necessary, assigned taxonomic names (i.e. NCBI taxonomy IDs) were manually corrected and curated to contain one taxonomic assignment per oligonucleotide. For probes targeting multiple taxa (e.g. two different species), we chose either the taxon that is predominantly covered by the probe or the next higher taxonomic rank that included all taxa (e.g. the genus, family, order, phylum or domain). Probe entries for which the specificity field did not contain any (e.g. ‘clone XYZ’) or only limited taxonomic information (e.g. ‘deltaproteobacterial symbiont of…’) were assigned to the root in the NCBI taxonomy or to the lowest meaningful taxonomic rank. The new taxonomy field in the probe details view shows the entire taxonomic hierarchy—from the assigned taxon to its highest taxonomic rank. It is noteworthy that this taxonomic assignment does not necessarily mean that an oligonucleotide is highly specific for a given taxon. Instead, it represents a systematic classification for all oligonucleotides in probeBase and allows for more advanced searches.

## SEARCH probeBase

**Figure 1.**
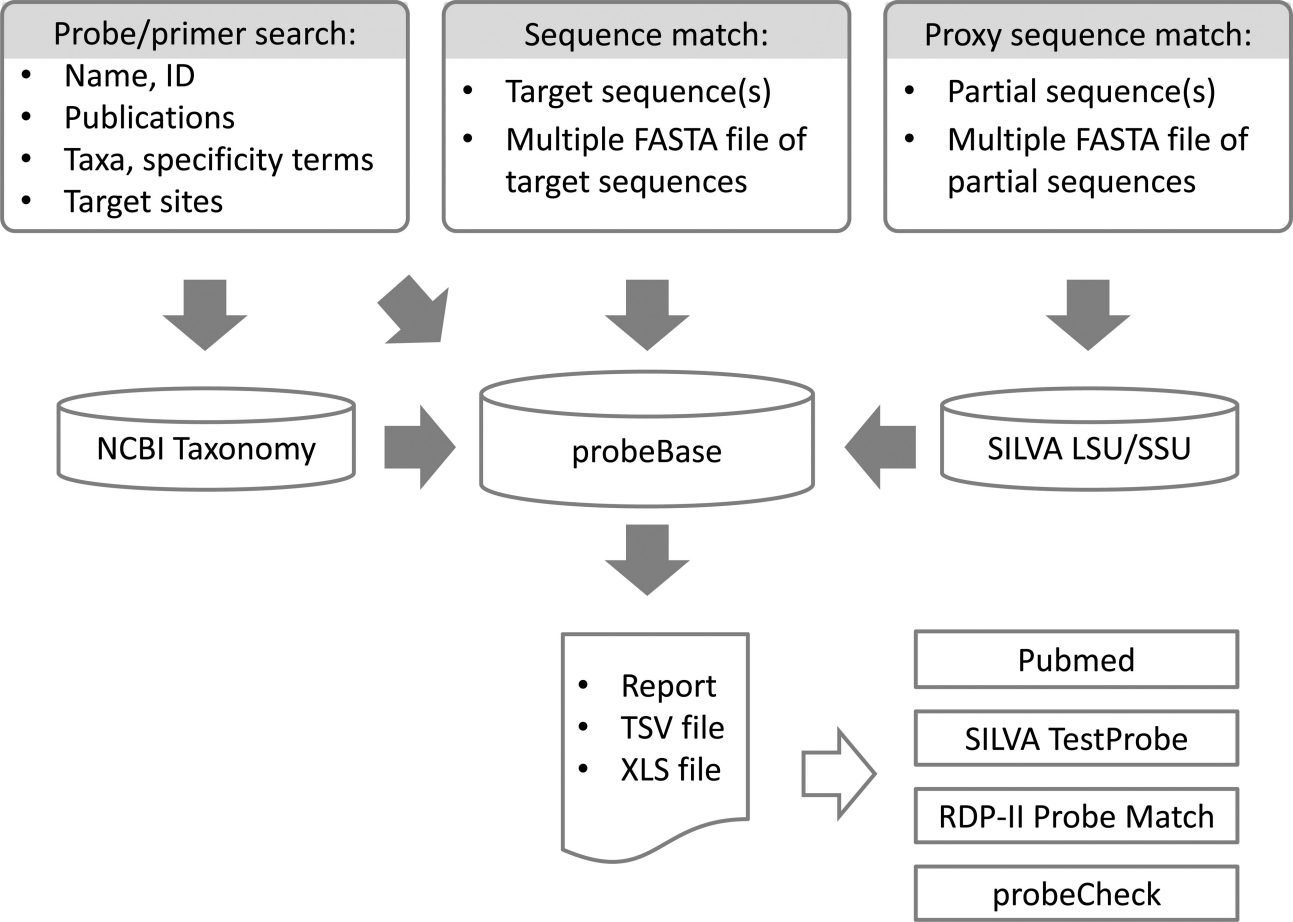
Structure and main tools of the probeBase database.

The ‘Search’ tool comprises multiple options for finding appropriate oligonucleotides and further information (Figure 1). Oligonucleotides can be recovered by the name of the target organism or taxon, by their specific target sites on the rRNA molecule, by the reference that originally described the oligonucleotide or simply by the name or sequence of the oligonucleotide itself. The output list of oligonucleotides that matched the search criteria can be restricted to primers, probes used successfully for FISH, and probes used on microarrays.

Because each probe is now assigned to a taxon in the NCBI taxonomy (20), a search for a specific target organism or taxon systematically returns all available oligonucleotides. The search target organism option is facilitated by an auto-complete function for taxonomic names to minimize the probability of typing errors. The search target organism option can be further adjusted by including oligonucleotides that target higher and/or lower taxonomic ranks than the query taxon. The corresponding output list of probes that target the query organism at different taxonomic levels helps researchers in identifying sets of hierarchically nested probes for application in multiple-probe hybridization formats such as multi-color FISH (10,12) and DNA microarrays (23).

The search for a probe/primer sequence not only yields perfectly complementary hits, but also oligonucleotides in probeBase with up to two mismatches to the query oligonucleotide. This allows, for example, to identify if a newly designed rRNA-targeted oligonucleotide and/or closely related variants of it have already been developed, tested for FISH and published before.

## MATCH rRNA QUERY SEQUENCES AGAINST OLIGONUCLEOTIDES IN probeBase

The original sequence match tool was developed to find all oligonucleotides in probeBase that perfectly match to up to 150 query rRNA sequences (14). However, due to the increased database size this tool was not usable and thus disabled for the past years. We have reimplemented and redesigned the sequence match tool (Figure 1), which is now based on a similarity search and able to process up to 1000 query sequences. Users can paste a (multi) fasta file into the text field, upload a (multi) fasta file or combine both options to query probeBase for complementary probes or primers with up to two mismatches. Results are either grouped by oligonucleotide, showing all matching sequences from the query per oligonucleotide, or by query sequence, showing all matching oligonucleotides per query sequence. A typical application of this tool is to quickly retrieve a set of available FISH probes that target rRNA sequences determined in an environmental microbial diversity survey without the need for extensive comparative sequence analysis. This probe set can then be readily applied for FISH to determine the abundance and spatial organization of the target organism in the sample (24).

## FIND FULL-LENGTH PROXY SEQUENCES FOR SHORT rRNA READS AND MATCH AGAINST OLIGONUCLEOTIDES IN probeBase

Traditional surveys of microbial diversity by PCR amplification of near full-length rRNA gene sequences from environmental DNA, cloning and Sanger sequencing of clone inserts have been almost completely replaced by highly parallel next generation sequencing of rRNA gene amplicons because of higher sample throughput and sequencing depth (i.e. number of sequences per sample). However, common technologies for multiplexed amplicon sequencing, such as Illumina MiSeq, produce only short (paired-end) reads that are typically less than 500 nucleotides in length. The short rRNA reads limit the selection of complementary oligonucleotides (e.g. by the ‘Match’ tool) for follow-up hybridization applications (25). Here, we provide a new ‘Proxy’ tool that finds corresponding near full-length rRNA sequences for short query sequences (Figure 1). These long proxy sequences are retrieved from the small or large subunit rRNA reference database of SILVA (17) and matched against the oligonucleotides in probeBase analogous to the ‘Match’ tool. The output includes the identified proxy sequences for each short query sequence and shows the probes or primers that have up to two mismatches to the proxy sequences.

## SUBMISSION OF MISSING OR NEWLY DEVELOPED OLIGONUCLEOTIDES

New or missing oligonucleotides can be submitted using an online form. The reference details (e.g. journal, authors, title, abstract, year) will now be automatically filled in by entering the PubMed-ID (PMID) (26) of a publication that contains new or missing probes/primers.

## AVAILABILITY

probeBase is maintained by the Department of Microbiology and Ecosystem Science, University of Vienna, Wien, Austria and available at http://www.probebase.net. We welcome comments concerning probeBase and highly appreciate reports of bugs, errors or missing probes. You may contact us by email to probebase@microbial-ecology.net.

## References

[1] Loy A., Lehner A., Lee N., Adamczyk J., Meier H., Ernst J., Schleifer K.-H., Wagner M. (2002). Oligonucleotide microarray for 16S rRNA gene-based detection of all recognized lineages of sulfate-reducing prokaryotes in the environment. Appl. Environ. Microbiol..

[2] DeAngelis K.M., Wu C.H., Beller H.R., Brodie E.L., Chakraborty R., DeSantis T.Z., Fortney J.L., Hazen T.C., Osman S.R., Singer M.E. (2011). PCR amplification-independent methods for detection of microbial communities by the high-density microarray PhyloChip. Appl. Environ. Microbiol..

[3] Muyzer G., de Waal E.C., Uitterlinden A.G. (1993). Profiling of complex microbial populations by denaturing gradient gel electrophoresis analysis of polymerase chain reaction-amplified genes coding for 16S rRNA. Appl. Environ. Microbiol..

[4] Amann R., Fuchs B.M. (2008). Single-cell identification in microbial communities by improved fluorescence in situ hybridization techniques. Nat. Rev. Microbiol..

[5] Herbold C.W., Pelikan C., Kuzyk O., Hausmann B., Angel R., Berry D., Loy A. (2015). A flexible and economical barcoding approach for highly multiplexed amplicon sequencing of diverse target genes. Front. Microbiol..

[6] Wu L., Wen C., Qin Y., Yin H., Tu Q., Nostrand J.D., Yuan T., Yuan M., Deng Y., Zhou J. (2015). Phasing amplicon sequencing on Illumina Miseq for robust environmental microbial community analysis. BMC Microbiol..

[7] Nemergut D.R., Costello E.K., Hamady M., Lozupone C., Jiang L., Schmidt S.K., Fierer N., Townsend A.R., Cleveland C.C., Stanish L. (2011). Global patterns in the biogeography of bacterial taxa. Environ. Microbiol..

[8] Dethlefsen L., Relman D.A. (2011). Incomplete recovery and individualized responses of the human distal gut microbiota to repeated antibiotic perturbation. Proc. Natl. Acad. Sci. USA.

[9] Seedorf H., Griffin N.W., Ridaura V.K., Reyes A., Cheng J., Rey F.E., Smith M.I., Simon G.M., Scheffrahn R.H., Woebken D. (2014). Bacteria from diverse habitats colonize and compete in the mouse gut. Cell.

[10] Behnam F., Vilcinskas A., Wagner M., Stoecker K. (2012). A straightforward DOPE (double labeling of oligonucleotide probes)-FISH (fluorescence in situ hybridization) method for simultaneous multicolor detection of six microbial populations. Appl. Environ. Microbiol..

[11] Pernthaler A., Pernthaler J., Amann R. (2002). Fluorescence in situ hybridization and catalyzed reporter deposition for the identification of marine bacteria. Appl. Environ. Microbiol..

[12] Valm A.M., Welch J.L.M., Rieken C.W., Hasegawa Y., Sogin M.L., Oldenbourg R., Dewhirst F.E., Borisy G.G. (2011). Systems-level analysis of microbial community organization through combinatorial labeling and spectral imaging. Proc. Natl. Acad. Sci. USA.

[13] Nikolakakis K., Lehnert E., McFall-Ngai M.J., Ruby E.G. (2015). Use of hybridization chain reaction-fluorescent in situ hybridization to track gene expression by both partners during initiation of symbiosis. Appl. Environ. Microbiol..

[14] Loy A., Horn M., Wagner M. (2003). probeBase: an online resource for rRNA-targeted oligonucleotide probes. Nucleic Acids Res..

[15] Loy A., Arnold R., Tischler P., Rattei T., Wagner M., Horn M. (2008). probeCheck - a central resource for evaluating oligonucleotide probe coverage and specificity. Environ. Microbiol..

[16] Cole J.R., Wang Q., Fish J.A., Chai B., McGarrell D.M., Sun Y., Brown C.T., Porras-Alfaro A., Kuske C.R., Tiedje J.M. (2014). Ribosomal Database Project: data and tools for high throughput rRNA analysis. Nucleic Acids Res..

[17] Quast C., Pruesse E., Yilmaz P., Gerken J., Schweer T., Yarza P., Peplies J., Glockner F.O. (2013). The SILVA ribosomal RNA gene database project: improved data processing and web-based tools. Nucleic Acids Res..

[18] Klindworth A., Pruesse E., Schweer T., Peplies J., Quast C., Horn M., Glockner F.O. (2013). Evaluation of general 16S ribosomal RNA gene PCR primers for classical and next-generation sequencing-based diversity studies. Nucleic Acids Res..

[19] Loy A., Maixner F., Wagner M., Horn M. (2007). probeBase–an online resource for rRNA-targeted oligonucleotide probes: new features 2007. Nucleic Acids Res..

[20] Federhen S. (2015). Type material in the NCBI Taxonomy Database. Nucleic Acids Res..

[21] Abouelhoda M.I., Kurtz S., Ohlebusch E. (2004). Replacing suffix trees with enhanced suffix arrays. J. Discrete Algorithms.

[22] Kielbasa S.M., Wan R., Sato K., Horton P., Frith M.C. (2011). Adaptive seeds tame genomic sequence comparison. Genome Res..

[23] Schönmann S., Loy A., Wimmersberger C., Sobek J., Aquino C., Vandamme P., Frey B., Rehrauer H., Eberl L. (2009). 16S rRNA gene-based phylogenetic microarray for simultaneous identification of members of the genus Burkholderia. Environ. Microbiol..

[24] Daims H., Lücker S., Wagner M. (2006). DAIME, a novel image analysis program for microbial ecology and biofilm research. Environ. Microbiol..

[25] Berry D., Schwab C., Milinovich G., Reichert J., Ben Mahfoudh K., Decker T., Engel M., Hai B., Hainzl E., Heider S. (2012). Phylotype-level 16S rRNA analysis reveals new bacterial indicators of health state in acute murine colitis. ISME J..

[26] Agarwala R., Barrett T., Beck J., Benson D.A., Bollin C., Bolton E., Bourexis D., Brister J., Bryant S.H., Canese K. (2015). Database resources of the National Center for Biotechnology Information. Nucleic Acids Res..

